# Chunky Mitral Annular Calcification: Caseoma or a Tumor?

**DOI:** 10.7759/cureus.58031

**Published:** 2024-04-11

**Authors:** Sakshi Khurana, Amit Gulati, Marlon E Rivera Boadla, Samuel Tan, Luka Katic, Anupam Sharma, Manish Vinayak, Kelash Kumar, Sachin Kumar, Amit Hooda

**Affiliations:** 1 Radiology, New York Presbyterian-Columbia University Irving Medical Center, New York, USA; 2 Cardiology, Icahn School of Medicine at Mount Sinai, New York, USA; 3 Internal Medicine, Maimonides Medical Center, Brooklyn, USA; 4 Internal Medicine, Icahn School of Medicine at Mount Sinai, New York, USA; 5 Hematology and Oncology, Fortis Hospital, Noida, IND; 6 Interventional Cardiology, Icahn School of Medicine at Mount Sinai, New York, USA

**Keywords:** atrial fibrillation, atrioventricular conduction, end stage renal disease (esrd), caseous calcification of the mitral annulus, mitral annulus calcification

## Abstract

Mitral annular calcification (MAC) is relatively common in clinical practice. Females are more often affected than males. Patients with end-stage renal disease have MAC relatively more commonly than the general population. Patients with MAC often develop conduction system disturbances, including advanced atrioventricular blocks. They are also more likely to develop various arrhythmias, including atrial fibrillation. Caseous mitral annulus calcification is a variant of MAC that often looks like a cardiac tumor on an echocardiogram and needs to be differentiated.

## Introduction

The mitral valve apparatus is composed of the left atrial wall, the left ventricular wall, the mitral annulus, the anterior and posterior leaflets, chordae tendineae, and papillary muscles. Mitral annular calcification (MAC) is a degenerative process affecting the mitral apparatus. It was initially thought of as an age-related process, but later, studies confirmed it to be an active process, with similarity to atherosclerosis and related calcification [1]. Several risk factors that are seen in patients with atherosclerosis like hypertension, hyperlipidemia, and end-stage renal disease (ESRD) are also common to MAC [1]. The posterior part of the mitral annulus is more commonly affected than the anterior annulus. 

Studies have shown that the prevalence of MAC is more common in females as compared to males [2]. Also, the incidence and prevalence increase with age. It is more common in patients with ESRD. Abnormal calcium and phosphate metabolism are likely the mechanism in patients with ESRD.

Transthoracic echocardiogram (TTE) is the most common and the cheapest modality for the diagnosis of MAC. It is also seen during fluoroscopy, especially in patients who are old and are on dialysis. Cardiac computed tomography (CT) along with magnetic resonance imaging (MRI) are superior imaging modalities for MAC as compared to TTE [3,4]. Conditions such as systemic hypertension, aortic stenosis, and hypertrophic cardiomyopathy, which increase stress on the mitral valve are risk factors for MAC. Left ventricle hypertrophy and mitral valve prolapse are commonly seen in patients with MAC. Caseous mitral annulus calcification (CMAC) is a variant of MAC that presents as an echo-dense mass with central echolucency. It simulates a periannular mass.

## Case presentation

A 63-year-old female was admitted to a tertiary care hospital for evaluation of a 1.2 mm melanoma lesion on her right arm. She was a diabetic and had hypertension along with hyperlipidemia. She had a history of cerebral aneurysm and had embolization done in 2014. She denied any cardiac disease in the past. She was a nonsmoker. During her preoperative evaluation, cardiology clearance was sought. Her labs were significant for hemoglobin of 8.8 mg/dL, hemoglobin A1C of 6.2%, and creatinine of 0.80 mg/dL.

**Figure 1:**
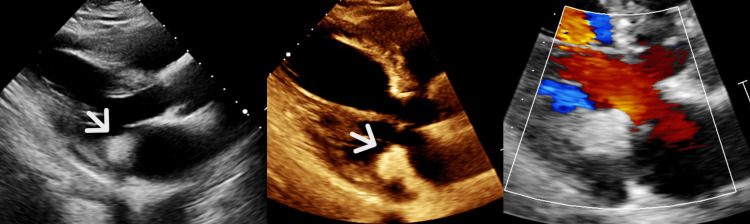
Parasternal long-axis view demonstrating an echogenic round lesion in the region of mitral annulus with mild posterior acoustic shadow (white arrow).

**Figure 2:**
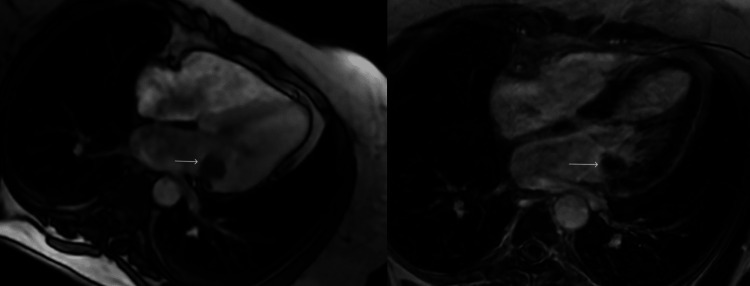
Axial T2 bright blood image and post-contrast T1-weighted image demonstrating a T2 hypointense non-enhancing focus of calcification corresponding to the echogenic focus seen on echocardiography (white arrow).

The electrocardiogram showed normal sinus rhythm with a normal axis. An echocardiogram was performed to rule out any structural heart disease and to assess for cardiac metastatic disease. The echocardiogram revealed an echogenic, nonmobile mass measuring 1.9 cm x 2.1 cm x 3.4 cm on the left ventricular side of the posterior mitral leaflet (Figure 1). Its appearance was most consistent with CMAC; however, other potential masses could not be excluded. The left atrium was moderately dilated. For further evaluation, a cardiac MRI with intravenous contrast was planned. On MRI, a T2 hypointense non-enhancing lesion was identified corresponding to the lesion noted on the echocardiogram, compatible with CMAC (Figure 2). The patient was cleared for surgery.

She remained asymptomatic throughout the hospital stay. She was planned for regular follow-up with cardiology as an outpatient.

## Discussion

A majority of patients with MAC are asymptomatic [5]. In most patients, it is an incidental finding discovered during a TTE conducted for a different indication. As discussed, MAC shares many risk factors with atherosclerosis like hypertension and renal failure. Various studies have confirmed the association between cardiovascular diseases (CVDs) and MAC. The prevalence of MAC was 8%-15% in patients without CVD, and it was high up to 42% in patients with CVD [6]. In the Framingham Heart Study, MAC was associated with increased cardiovascular death and all-cause mortality [7]. Jeon et al. demonstrated that patients with MAC have a higher prevalence of obstructive coronary artery disease [8]. There is a higher prevalence of stroke in patients with MAC [9,10]. All this demonstrated that MAC may be a marker of atherosclerosis.

Along with being a marker for atherosclerosis, MAC also causes local mitral valve dysfunction. The gradual progression of the disease to mitral valve leaflets and subvalvular apparatus can lead to varying degrees of mitral stenosis and mitral insufficiency. In the Euro Heart Study, in elderly patients with mitral stenosis, MAC was present in 60% of the patients [11].

MAC has been associated with calcific aortic stenosis as well. They share common pathophysiological mechanisms. In patients undergoing transcatheter aortic valve implantation, MAC was found in 9.5% of the patients. Infective endocarditis is relatively rare in patients with MAC, as reported by Minardi et al. [12].

Patients with MAC who also have mitral regurgitation or mitral stenosis and are undergoing mitral valve surgery have higher chances of undergoing mitral valve replacement rather than mitral valve repair. Surgery with calcium debridement can lead to left circumflex artery injury. If calcium debridement is avoided, it leads to a smaller annular diameter with resultant paravalvular regurgitation post-surgery. Complete annular dissection with reconstruction may be an option in these patients. Mitraclip may be an option along with transcatheter mitral valve replacement in high-risk patients for surgery.

MAC has been associated with various arrhythmias. It can be associated with atrial fibrillation, which is secondary to left atrium chronic changes due to mitral valve disease. Conduction disorders with varying degrees of atrioventricular blocks are also seen in patients with MAC. This may be secondary to a direct extension of calcification to the conduction system or primary conduction tissue disease. 

CMAC is a rare variant of mitral annular calcification [13]. CMAC is mostly present in the posterior mitral annulus. It's mostly seen on TTE as an echodense structure with central echolucency. The reason for the caseation is not well understood. It has been associated with systemic embolization likely from the calcium or ulceration and subsequent thrombus formation. CMAC can mimic vegetation, a tumor, or a cyst. Follow-up on patients with CMAC has shown that it can resolve spontaneously. Regular follow-up is needed for resolution or evidence of the progression of CMAC. It's medically managed unless it impedes left ventricular filling or obstructs left atrial outflow. Anticoagulation is indicated if there is evidence of thrombus associated with MAC and if it embolizes.

## Conclusions

MAC is an active process similar to atherosclerosis, sharing many common risk factors. It's mostly diagnosed incidentally in patients getting a TTE for a different reason. CMAC is a rare variant, which can simulate an annular mass. It is medically managed and may resolve spontaneously unless it causes left atrial obstruction. Surgery for mitral valve dysfunction may need modification of the surgical technique. Anticoagulation is indicated for patients who have embolic episodes. MAC has been shown in various studies to be associated with all-cause mortality.
